# Penalized eigenvalue block averaging: Extension to nested model comparison and Monte Carlo evaluations

**DOI:** 10.3758/s13428-026-02968-4

**Published:** 2026-04-08

**Authors:** Njål Foldnes, Steffen Grønneberg, Jonas Moss

**Affiliations:** 1 Norwegian Centre for Reading Education and Research, University of Stavanger, Stavanger, Norway; 2 Department of Economics, BI Norwegian Business School, Oslo, Norway; 3 Department of Data Science and Analytics, BI Norwegian Business School, Oslo, Norway

**Keywords:** Goodness-of-fit test, Nested model comparison, Measurement invariance, Factor model, Non-normality, pEBA

## Abstract

Testing goodness-of-fit and multi-group nested models in confirmatory factor analysis under non-normality is foundational in psychometrics and related fields. Recently, a penalized eigenvalue block averaging (pEBA) procedure was proposed for testing goodness-of-fit, showing promise in a restricted type I error control simulation study. In this study, we extend the simulation conditions to higher dimensions for latent and observed vectors and evaluate type I error control and power for many pEBA variants and traditional test statistics. All statistics are evaluated in four versions, by crossing the base statistic (ML or Browne’s RLS), and whether the asymptotic covariance matrix estimator was bias-corrected or not. We develop pEBA methods in the new setting of nested model comparison, accompanied by extensive Monte Carlo evaluation of their performance in weak invariance testing, including type I error control and power. The best-performing procedure for goodness-of-fit testing was pEBA with four blocks, based on the RLS statistic, using the asymptotic covariance matrix estimator without bias correction. For measurement invariance, pEBA with singleton blocks, using the standard ML statistic and the unbiased estimator for the asymptotic covariance matrix, performed best. The pEBA procedures are available in the newly developed R package semTests.

Researchers in the social and behavioral sciences routinely examine whether latent psychological constructs may explain the observed covariances among scale items. To do so, a central assessment tool is the chi-square goodness-of-fit test associated with a factor model that relates latent constructs to scale items. If the test suggests that the model is correctly specified, a further essential requirement on the scale is that its measurement properties do not vary across groups and measurement occasions. If this is the case, measurement invariance is said to hold (see, e.g., Meredith, 1993; Millsap, 2012).

Historically, test statistics were developed under the assumption of multivariate normality. These tests perform well under ideal conditions where a large random sample is drawn from a multivariate normal distribution. However, it is well known that non-normal data are ubiquitous in real-world datasets (Cain et al., 2017; Micceri, 1989). For such data, tests based on normal theory generally fail to maintain type I error control and typically reject well-specified models more often than stipulated by the significance level (e.g., Curran et al., 1996).

Consequently, robustified test procedures, such as the mean-scaling procedure of Satorra and Bentler (1988) and the scaling and shifting procedure of Asparouhov and Muthén (2010), have been developed to lessen the overrejection problem. These tests are currently the default choices in software packages such as lavaan (Rosseel, 2012) and Mplus (Muthén & Muthén, 2017), and are consequently used extensively in social science research. However, although these tests mitigate the declining performance of normal-theory based test procedures under non-normality, their respective performances have been shown in many conditions to be sub-optimal (e.g., Foldnes & Grønneberg, 2018; Foldnes et al., 2025; Foldnes & Olsson, 2015), with empirical rejection rates often far removed from the nominal level of significance. Consequently, in recent years, new test procedures have been proposed in order to find a test with a better type I control than the classical robustified tests (Foldnes & Grønneberg, 2018; Foldnes et al., 2025; Hayakawa, 2019; Wu & Lin, 2016). In particular, Foldnes et al. (2025) identified new test procedures, referred to as penalized eigenvalue block averaging (pEBA) tests, that outperformed established robustified tests when evaluating the goodness-of-fit of factor models with up to 40 observed variables.

The contributions of our article are as follows. Given the critical importance of model fit testing in confirmatory factor analysis (CFA), and the lack of consensus regarding the choice of test statistic under non-normality, it is important to further investigate whether the pEBA approaches may be preferable to established tests. Goodness-of-fit model testing using pEBA and established tests was the focus of Foldnes et al. (2025). Here, we substantially expand the simulation conditions of Foldnes et al. (2025) to higher dimensions. Given the novelty of pEBA methods, it is unclear whether their superiority extends to higher dimensions. In addition, we obtain power results for pEBA, a topic that has not been investigated in the literature. Under our extended simulation conditions, we find support for the conclusions of Foldnes et al. (2025), and find that the pEBA methods retain their superiority over established tests, while exhibiting acceptable power.

Another procedure of critical importance in factor analysis is multi-group nested model comparisons, for instance, in measurement invariance testing. Motivated by the promising initial performance of pEBA in single-model testing, we here theoretically develop its use for nested model comparisons. To evaluate whether pEBA methods can outperform established tests in the context of nested model testing, we conduct simulation studies for the first time to investigate type I error control and power. For our evaluation, we focus on measurement invariance testing across groups, a topic of high practical importance (Chapter 1 Millsap, 2012). Specifically, we investigate weak (metric) invariance–typically the first substantive checkpoint in an invariance sequence (Chapter 4 Millsap, 2012). In addition, many other test statistic variants, hitherto unexamined in the context of measurement invariance testing, are evaluated. Our findings suggest that some pEBA variants outperform commonly used established tests in terms of type I error control and power.

A final contribution is the R (R Core Team, 2025) package semTests (Moss, 2024), which has been extended to handle nested model comparisons such as measurement invariance testing.

This article is organized as follows. In the next section, we define the test procedures under study and discuss relevant literature on their performance. Then we give the rationale for conducting our present study, and extend the pEBA procedures to handle multi-group nested model comparison. This is followed by sections containing methods and results. The article concludes with a discussion of our findings, their limitations, ideas for future research, and practical recommendations. Supplementary material in the form of code, complete simulation specifications, and simulation results are found at https://osf.io/h2y3n/, with an accompanying guide in *readme.pdf*.

## Tests of goodness-of-fit in confirmatory factor models

We review the asymptotic testing framework for confirmatory factor models, as described by, e.g., Satorra (1989). Suppose we randomly draw *N* observations from a *p*-dimensional random vector $x=(x_1, \ldots , x_p)$. The confirmatory factor model (see Bollen, 1989, for a textbook introduction) connects *x* and an *l*-dimensional vector of latent factors *f* through the linear equation

1 $$\begin{aligned} x=\Lambda f + \epsilon , \end{aligned}$$

where we, for ease of presentation, assume that *x* has a zero population mean. Here, ${\epsilon }$ is a *p*-dimensional vector of mean-zero residuals that are uncorrelated with *f*. Then,

2 $$\begin{aligned} \Sigma = {\text {Cov}} (x) = \Lambda \Phi \Lambda ' + \Psi , \end{aligned}$$

in which $\Phi = {\text {Cov}} (f)$, and $\Psi = {\text {Cov}} (\epsilon )$. Under additional restrictions on $\Lambda , \Phi$, and $\Psi$ (Bollen, 1989, Chapter 7), the model is identified. We collect the *q* free parameters into a vector $\theta$, and write the model-implied covariance matrix as $\Sigma (\theta ) = \Lambda \,\Phi \,\Lambda ' + \Psi$.

The dominant estimation methodology for confirmatory factor models (Bollen, 1989, Chapter 7) is the normal-theory maximum likelihood (NTML) estimator $\hat{\theta }_\text {NTML}$ that minimizes

3 $$\begin{aligned} \theta \mapsto F_\text {NTML} \left( S, \Sigma (\theta ) \right) =\ln {|\Sigma (\theta )|} + {{\,\textrm{tr}\,}}\big ( S \Sigma (\theta )^{-1}\big ) - \ln {|S|}-p, \end{aligned}$$

where *S* is the biased sample covariance matrix and *p* is the dimension of *x*. In this article, this is the only estimation method we consider.

### Testing goodness-of-fit in confirmatory factor models

The null hypothesis of a correctly specified model versus the alternative hypothesis of model misspecification is most commonly tested using the normal-theory fit statistic

4 $$\begin{aligned} T_\text {ML}= (N-1)F_\text {NTML}(S, \Sigma (\hat{\theta }_\text {NTML})).\end{aligned}$$

We also consider the reweighted least squares (RLS) fit statistic (Browne, 1974, equation  (24), p. 7)

5 $$\begin{aligned} T_\text {RLS}= \frac{N}{2} {{\,\textrm{tr}\,}}\left( [I - \Sigma (\hat{\theta }_\text {NTML})^{-1} S]^2 \right) , \end{aligned}$$

which converges in distribution to the same limit as $T_\text {ML}$ (Browne, 1974, Propositions 6 & 7). When the observed variables are normal, the limit is a chi-square distribution with $d=p(p+1)/2-q$ degrees of freedom,

6 $$\begin{aligned} T_\text {ML}\xrightarrow [N \rightarrow \infty ]{D} \sum _{j=1}^d Z_j^2, \qquad Z_1, \ldots , Z_d \sim N(0,1) \text { IID}. \end{aligned}$$

In recent Monte Carlo studies, under normality, the *p* value based on $T_\text {RLS}$ performed better than the *p* value based on $T_\text {ML}$ (Foldnes et al., 2025; Hayakawa, 2019; Zheng & Bentler, 2022).

With potentially non-normal data, Satorra and Bentler (1986) showed (see also Satorra & Bentler, 1990, Eq. (2.13a), p. 239) that the limit distribution becomes a weighted sum of squares instead

7 $$\begin{aligned} T_\text {ML}\xrightarrow [N \rightarrow \infty ]{D} \sum _{j=1}^d \lambda _j Z_j^2, \qquad Z_1, \ldots , Z_d \sim N(0,1) \text { IID}, \end{aligned}$$

where the weights $\lambda = (\lambda _1, \ldots , \lambda _d)$ are positive eigenvalues of the matrix $U \Gamma$. Here $\Gamma$ is the asymptotic covariance matrix of sample covariances and depends only on the multivariate distribution of the data, and

$$\begin{aligned} U={V}- {V} \Delta \left\{ \Delta ' {V} \Delta \right\} ^{-1}\Delta '{V} \end{aligned}$$

is a matrix depending only on the model, where $\Delta$ is the derivative matrix of the half-vectorization $\sigma (\theta )$ of $\Sigma (\theta )$. Also, $V = \frac{1}{2} D_p' (\Sigma (\theta )^{-1} \otimes \Sigma (\theta )^{-1}) D_p$, where $D_p$ is the duplication matrix (Magnus & Neudecker, 2019, ch. 3.9).

There are combinations of models and non-normal distributions where the weights $\lambda _j$ are all equal to one. In these cases, inference is said to be asymptotically robust (Satorra & Bentler, 1990, Section 3) in the sense that $T_\text {ML}$ has the usual $\chi ^2$ limit of equation (6). Our interest is in distributions where inference is not asymptotically robust. If the weights $\lambda _j$ are not equal to one, the standard chi-square *p* value based on equation (6) is incorrect, even in large samples. To correct for this, the weights $\lambda _j$ must be estimated and incorporated into the *p* value calculation based on the $\chi ^2$-mixture of equation  (7). We estimate $\hat{\lambda }$ using the *d* positive eigenvalues of $\hat{U} \hat{\Gamma }$, where $\hat{U}$ and $\hat{\Gamma }$ are estimators of *U* and $\Gamma$. For *U*, we use the standard plug-in estimator, replacing population parameters by their empirical estimates (see, e.g., Satorra, 1989, Section 2). For $\Gamma$, we consider two alternatives. The first, which is the default in most software packages, replaces population expectations in Browne’s formula (Browne, 1984, equation 3.4) with sample averages. The second, which was recently studied by Du and Bentler (2022), is an unbiased modification of the first estimator (Browne, 1984, equation 3.8).

### Eigenvalue-based goodness-of-fit tests

The vector $\hat{\lambda }$ is an unstable estimator of the *d*-positive eigenvalues of $U \Gamma$, even in large samples (Foldnes et al., 2025, Section 1.4, see also Table 5, p. 11). This motivates transforming $\hat{\lambda }$ into a stabilized approximation, which we call $\tilde{\lambda }$. An important example of such a stabilizing approximation is the Satorra–Bentler (SB) correction (Satorra & Bentler, 1988). The Satorra–Bentler correction is usually presented as a rescaling of $T_\text {ML}$, but it can also be represented (Foldnes et al., 2025, Eq. (5), p. 3) as

8 $$\begin{aligned} \tilde{\lambda }_i = \frac{1}{d} \sum _{j=1}^d \hat{\lambda }_j, \quad i=1, 2, \ldots , d. \end{aligned}$$

So instead of using $\hat{\lambda }$ directly, the empirical average is used as a stabilized approximation to each weight.

Based on an estimated $\tilde{\lambda }$, we may calculate *p* values as follows. Consider the function *H* defined by

9 $$\begin{aligned} H(t ; l_1, \ldots , l_d) = P \left( \sum _{j=1}^d l_j Z_j^2 > t \right) , \qquad Z_1, \ldots , Z_d \sim N(0,1) \text { IID}. \end{aligned}$$

Let $\tilde{H}(t) = H(t; \tilde{\lambda }_1, \ldots , \tilde{\lambda }_d)$. Then the *p* values of the goodness-of-fit test based on either $T_\text {ML}$ or $T_\text {RLS}$ are

10 $$\begin{aligned} \tilde{H}(T_\text {ML}) \text { and } \tilde{H}(T_\text {RLS}), \end{aligned}$$

respectively.

If the stabilizing step is skipped, we may input the raw estimate $\tilde{\lambda }=\hat{\lambda }$ into *H* to produce a *p* value. This procedure, which we call ALL, was investigated by Foldnes and Grønneberg (2018, under the name EBAd), and also by Jobst et al. (2022); Wu and Lin (2016). The eigenvalue block averaging (EBA) procedures proposed by Foldnes and Grønneberg (2018) extend the Satorra–Bentler approach by using $\tilde{\lambda }$ defined as averages of equally sized blocks of consecutive eigenvalues in $\hat{\lambda }$, which is assumed to be sorted in increasing order. The penalized eigenvalue block averaging (pEBA) procedures were proposed by Foldnes et al. (2025) to improve the performance of EBA by averaging the grand average and the block averages. pEBA with *x* blocks is denoted by pEBAx. Foldnes et al. (2025) also considered estimates $\tilde{\lambda }$ obtained from a penalized simple linear regression estimate on $\hat{\lambda }$ (sorted in increasing order) against the index $i=1, 2, \ldots , d$. The resulting procedure is named pOLS2.

### The scaled and shifted test and the scaled *F*-test

The scaled and shifted (SS) test of Asparouhov and Muthén (2010) scales and shifts the test statistic *T* under consideration (here, either $T_\text {ML}$ or $T_\text {RLS}$), via the formula $T_\text {SS}=a T+d-b,$ where $a=\sqrt{d/\text {tr}\left( (\hat{U} \hat{\Gamma })^2\right) }$ and $b=\sqrt{d \left( \text {tr}( \hat{U} \hat{\Gamma }) \right) ^2 / \text {tr}\left( (\hat{U} \hat{\Gamma })^2\right) }$. The *p* value for the test is then

11 $$\begin{aligned} P (\chi ^2_d > t)_{t = T_\text {SS}}. \end{aligned}$$

Under the null hypothesis, $T_\text {SS}$ has the same expectation and variance as a $\chi ^2_d$ distribution in the limit. Foldnes and Olsson (2015) found that SS underrejected correctly specified models, while Foldnes et al. (2025) found that procedures pEBAx, pOLS, and Satorra–Bentler (SB) outperformed SS in terms of type I error control.

The scaled *F*-test (SF) of Wu and Lin (2016) uses a scaled *F* distribution to approximate the distribution of $T_\text {ML}$. Both the scaling factor, and the two degrees of freedom of the *F* distribution are functions of $\sum _{i=1}^d \hat{\lambda }_i$, $\sum _{i=1}^d \hat{\lambda }_i^2$, and $\sum _{i=1}^d \hat{\lambda }_i^3$. Foldnes and Grønneberg (2018) found its performance to be quite similar to that of SS when simulating from a structural equation model with 12 observed variables.

### New variants of established procedures: The RLS and UG variants of tests

Following Du and Bentler (2022) and Foldnes et al. (2025), all test procedures may be calculated by estimating $\Gamma$ both with and without bias correction. Additionally, following Foldnes et al. (2025), each procedure may be calculated with both $T_\text {ML}$ and $T_\text {RLS}$, as in equation (10).

The procedures are denoted by a subscript RLS if $T_\text {RLS}$ is used, and a superscript UG if the unbiased $\Gamma$ estimator is used. For example, $\text {SB}_\text {RLS}^\text {UG}$ is the SB test based on $T_\text {RLS}$ and using the unbiased $\Gamma$ estimator, while $\text {SB}^\text {UG}$ is the SB test based on $T_\text {ML}$ and the unbiased $\Gamma$ estimator.

## Extending pEBA to multi-group nested model testing, with applications to measurement invariance testing

We extend the pEBA procedures to multi-group nested model comparison. Satorra (2000, Section 3) shows that standard nested model comparison test statistics have a weighted sum of chi-squares limit similar to that of equation (7). We may use this to develop pEBA procedures for nested model comparison supporting multiple groups, as needed in invariance testing. The required numerical work is implemented in the accompanying R package semTests (Moss, 2024).

We outline the inference theory underlying the extension of pEBA to nested tests. For conciseness, we do not review multiple group analyses in full, and instead refer to, e.g., Millsap (2012, Chapter 4) for estimation theory. Briefly, *G* independent datasets are sampled, giving observations from groups $g=1, 2, \ldots , G$. Within each group, the sampled observations follow a confirmatory factor model, giving a covariance matrix as in equation (2), though with matrices $\Lambda _g, \Phi _g, \Psi _g$ possibly varying with *g*. These matrices, when combined, result in a joint covariance model.

We consider two models, $M_0$ with $d_0$ degrees of freedom, and $M_1$ with $d_1$ degrees of freedom, where $M_0$ is nested within $M_1$, subject to the technical conditions described in Satorra (2000, Section 3), which rules out certain degenerate cases. These conditions are fulfilled, for example, when $M_1$ is such that the factor loadings in $\Lambda _g$ may vary freely among groups, while in $M_0$ they are constrained to be equal among groups. This situation is the one examined in weak measurement invariance testing, for which we conduct an extensive Monte Carlo study. Let $T_0$ and $T_1$ both be either $T_\text {ML}$ or $T_\text {RLS}$, calculated under $M_0$ and $M_1$, respectively. The null hypothesis is that $M_0$ suffices, while the alternative hypothesis is that $M_1$ is needed. Consider the test statistic

12 $$\begin{aligned} T_D = T_0 - T_1, \end{aligned}$$

which increases to infinity when $N \rightarrow \infty$ under the alternative hypothesis. Under the null, and when using $T_\text {ML}$ with normal data, this is the log-likelihood ratio statistic, which is asymptotically $\chi ^2$-distributed with $d_0 - d_1$ degrees of freedom.

Under the null, and under non-restrictive distributional assumptions of Satorra (2000, Section 3), $T_D$ converges to a weighted sum of chi-square distributions, as was the case for goodness-of-fit tests:

13 $$\begin{aligned} T_D \xrightarrow [N \rightarrow \infty ]{D} \sum _{i=1}^{d_0-d_1} \lambda _i Z_i^2. \end{aligned}$$

Here, $\lambda _i$ are the eigenvalues of a matrix $U_D \Gamma$, where $\Gamma$ is the same matrix as in the goodness-of-fit case, while $U_D$ is a matrix that depends only on the population parameter vector of $M_1$, and is estimated using the corresponding parameter estimates (see Satorra, 2000, equation (22)). The matrix $U_D \Gamma$ has $d_0 - d_1$ positive eigenvalues, the rest being zero.

### Two ways of estimating $U_D$

We consider two ways of computing $U_D$. In Satorra (2000, equation (22)), a formula is given for $U_D$, which we refer to as the 2000 method. An alternative formula is given in Satorra and Bentler (2001, p. 510), where $U_D$ is computed as the difference of $U_0, U_1$ where $U_0, U_1$ are the *U*-matrices of the models $M_0$ and $M_1$. This formula we refer to as the 2001 method. Satorra and Bentler (2001, p. 510) sketch a proof of the equality $U_D = U_0 - U_1$, but at least in finite samples, considerable differences between the two ways of computing $U_D$ can arise, as the 2000 method relies on parameters estimated under $M_1$ only, while the 2001 method uses parameter estimates from $M_0$ for $U_0$ and parameter estimates from $M_1$ for $U_1$.

The original formulation of the SB test is based on the trace of an estimate of $U_D \Gamma$. In the population, this equals the sum of the eigenvalues of $U_D \Gamma$. In the 2001 method, the trace and the sum of the $d_0 - d_1$ eigenvalues may differ considerably for a given dataset, even under the null hypothesis. In the semTests package, we compute the SB test using the sum of the $d_0 - d_1$ eigenvalues of the estimated $U_D \Gamma$. Using the 2000 method for $U_D$ gives the same number up to numerical precision as the trace of the estimated $U_D \Gamma$. In contrast, with the 2001 method for $U_D$, the two calculation methods may differ significantly. Due to this difference, we additionally include in our upcoming simulations the SB test with the 2001 method as implemented in lavaan to assess the original SB formula suggested by Satorra and Bentler (2001) in the simulations.

Given that the 2001 method can result in negative test statistic values, Satorra and Bentler (2010) proposed a modified method that ensures a non-negative test statistic value. The 2010 method involves the additional step of fitting $M_1$ using the $M_0$ estimates as fixed parameters. Chuang et al. (2015) found in nested confirmatory factor models that the 2010 and 2001 methods tended to over- and underreject the null, respectively. Brace and Savalei (2017) evaluated the 2001 and 2010 methods in the context of two-group measurement invariance testing, and found the 2010 method to be slightly superior to the 2001 method. Pavlov et al. (2020) also included the SS test in a nested confirmatory factor model comparison study, and found that it outperformed both SB with method 2001 and SB with method 2010. The latter two tests performed similarly in Pavlov et al. (2020).

To the best of our knowledge, nested model comparisons based on the formulas in Satorra (2000) have not yet been evaluated. This omission might be due to the unavailability of software implementations. Variants based on the 2001 method are evaluated by Brace and Savalei (2017).

## The present study

Recently, Foldnes et al. (2025) proposed a new class of test statistics for structural equation modeling under non-normality, based on penalizing the EBA procedures of Foldnes and Grønneberg (2018). In a simulation study, Foldnes et al. (2025) found that the new penalized procedures outperformed established tests such as SB and SS in confirmatory factor models with two factors. As shown in the previous section, these procedures also apply to nested model comparisons, which were neither considered nor evaluated in Foldnes et al. (2025).

Goodness-of-fit testing and nested model comparisons under non-normality are extensively applied in the social sciences. It is therefore of interest to further investigate the penalized procedures and provide researchers with recommendations regarding which test procedures best control type I error rates and attain adequate power in practical settings. Our study considerably extends the simulation settings in Foldnes et al. (2025). Whereas Foldnes et al. (2025) considered only type I error rates, two factors, and data dimensions of 10, 20 or 40, we consider both type I error rates and statistical power, consider a five-factor model, and data dimensions of 15, 30, 60, and 100. To keep these results directly comparable with Foldnes et al. (2025), we use the same distributional classes as them. Moreover, we conduct the first Monte Carlo evaluation of the penalized EBA procedures for nested model comparisons in the form of testing for weak measurement invariance. Measurement invariance is evaluated both in terms of type I error control and power.

In addition to studying the performance of new procedures, we also include the previously mentioned new versions of established tests in our investigation. For instance, the established SB test has been extensively studied in several Monte Carlo investigations (e.g., Curran et al., 1996; Foldnes & Olsson, 2015). However, most of these studies consider only the version based on $T_\text {ML}$ and the biased $\Gamma$ estimator, since these are the defaults in software packages. Exceptions are Hayakawa (2019), who also includes $T_\text {RLS}$, and Du and Bentler (2022), who employ the generalized least squares estimator and fit statistic, paired with both biased and unbiased $\Gamma$ estimators, in factor models with up to 30 observed variables. In the present study, we employ normal-theory maximum likelihood estimation, and for each established test, we fully cross $T_\text {ML}$ vs. $T_\text {RLS}$ with unbiased vs. biased $\Gamma$ estimation, to obtain four versions for each of the test procedures.

For nested model comparisons, several studies (e.g., Brace & Savalei, 2017; Pavlov et al., 2020) evaluate chi-square difference tests for SB and SS, based on the approach of Satorra and Bentler (2001), who simplify the scaling procedure proposed in Satorra (2000). The latter 2000 method was not available in standard software until 2015, in version 0.5-18 of the R package lavaan (Rosseel, 2012), explaining the lack of studies comparing the 2000 method with the 2001 method. In the present study, we comprehensively evaluate and compare these two methods.

## Method

Three Monte Carlo studies were conducted to determine the performance of test procedures. In Study 1, the goodness-of-fit testing of the confirmatory factor models was evaluated under correct model specification, while Study 2 evaluated nested model comparisons in multigroup measurement invariance testing under correct model specification. Study 3 evaluated power to detect misspecified models for both goodness-of-fit testing and nested model comparisons.

### Simulation conditions common across the three studies

*Sample size*: To reflect a range of sample sizes commonly encountered in empirical work, we considered sample sizes $n=400, 800,$ and 2000. For weak invariance testing, these sample sizes apply at the group level, giving a larger total sample size depending on the number of groups.

*Distributions*: As discussed by Fairchild et al. (2024), test performance depends on the distributional aspects of the data. To increase the external validity of our findings, we therefore considered four classes of distributional families: the normal distribution, the Vale–Maurelli (VM) (Vale & Maurelli, 1983) distribution, the independent generator (IG) distribution (Foldnes & Olsson, 2016), and the piecewise linear (PL) distribution (Foldnes & Grønneberg, 2022). For each of the latter three non-normal distributional classes, ML inference is known to not be asymptotically robust (Foldnes et al., 2025), and for each distributional class we included two members, based on the univariate skewness and kurtosis of the marginals. So in total, seven distributions were considered, which we denote by N, VM1, VM2, IG1, IG2, PL1, and PL2. Here, VM1, IG1, and PL1 have moderate (Curran et al., 1996, p. 20) marginal skewness and kurtosis with values 2 and 7, respectively, and VM2, IG2, and PL2, have severe (Curran et al., 1996, p. 20) marginal skewness and kurtosis, with values 3 and 21, respectively. It is important to note that although the covariance matrix and marginal skewness and kurtosis of, e.g., VM1, PL1, and IG1, are identical, the three distributions are not identical, with not even identical marginal distributions.

We verified that the observed skewness and kurtosis converged to population targets with increasing sample size. At the smallest size, $n=400$, observed values fell noticeably below targets, consistent with the well-known negative finite-sample bias of moment estimators (see online supplementary material for a full validation table).

*Population model parameters:* In the population model, all latent variables and error variances have unit variance and the factor loadings were randomly drawn from the uniform distribution in the range [0.5, 1.5]. This corresponds to standardized factor loadings in the interval [0.45, 0.83], which is commonly found in research practice (Li, 2016, p. 373). In both studies, the factor loadings in a smaller-dimensional model are nested within the larger-dimensional models. For a complete description of population models, see the online supplementary material.

### Test procedures

All tests were based on normal-theory ML parameter estimation. We included the normal-theory tests RLS and ML, and 36 robustified tests. The robustified tests were obtained from nine test methods: ALL, pEBA with 2, 4, 6, and *d* blocks, pOLS, SS, SB, and SF. For each method, four versions were calculated, yielding a total of 36 robustified tests: Either using the biased $\Gamma$ estimator or the unbiased $\Gamma$ estimator, and either using $T_\text {RLS}$ or using $T_\text {ML}$ as the base statistic.

For nested model comparisons, we include two versions of each of the 36 robustified tests, based on whether the $U_D$ matrix is calculated using the estimator proposed by Satorra (2000) or the estimator proposed by Satorra and Bentler (2001). Also, we included an additional test, namely the SB test, using the method of Satorra and Bentler (2001), which is calculated using the trace of $\hat{U}\hat{\Gamma }$, and is denoted by $\text {SB}_\text {2001}$. This test is the default model comparison procedure in lavaan for non-normal data under the estimation option “MLM”.

### Study 1: Goodness-of-fit for confirmatory factor models

We consider a five-factor model. Each factor has the same number of indicators, and the factor loadings are inspired by those found in empirical studies on the Big Five personality factors (e.g., McCrae & Costa, 2004, Table 1). While this parameter choice reflects parameter values relevant to similar applications, our data-generating mechanisms do not aim to, and are not capable of, realistically emulating Big Five personality data. The interfactor correlations take the values $-.3, -.2, 0,.1,.2,$ and .3 and are fully reported in the online supplementary material.

We include a range of model sizes with a total number of items equal to $p=15, 30, 60,$ and 100. The total number of simulation conditions obtained by fully crossing the three sample sizes, four dimensions, and seven distributions is 84.

Table 1 contains the dimensions, sample sizes, number of free parameters, and degrees of freedom for the four models. We also calculate the ratio of sample size to the number of free parameters to identify conditions with inadequate sample size. Kline (2015, p.16) refers to conditions where $n/q < 10$ as less than ideal. We label such conditions as having an inadequate sample size. The population item covariances and correlations for all model sizes are available in the online supplementary material.

**Table 1:** Study 1. Model size and sample size conditions

*p*	*n*	*q*	df	*n*/*q*	$n/q \ge 10$
15	400	40	80	10	1
15	800	40	80	20	1
15	2000	40	80	50	1
30	400	70	395	5.7	0
30	800	70	395	11.4	1
30	2000	70	395	28.6	1
60	400	130	1700	3.1	0
60	800	130	1700	6.2	0
60	2000	130	1700	15.4	1
100	400	210	4840	1.9	0
100	800	210	4840	3.8	0
100	2000	210	4840	9.5	0

*Note.* *p* = number of observed variables. *n* = sample size. *q* = number of free model parameters

### Study 2: Weak measurement invariance testing

In this study, we investigate testing for weak invariance, i.e., we test whether factor loadings are invariant across groups: $\Lambda =\Lambda _g$ for all groups *g*. Invariance testing in the context of confirmatory factor models is mainly used to assess whether psychological constructs are measured equivalently across population groups (Millsap, 2012, Chapter 4).

We consider a one-factor model with three model sizes: the number of items is $p=5, 10$, or 20. The number of groups was set to 2, 4, or 8.

The total number of simulation conditions obtained by fully crossing the three sample sizes, three dimensions, three group sizes, and seven distributions is 189. Table 2 gives for each combination of *p*, *n*, and number of groups, the number of parameter constraints *q* and the degrees of freedom $df_1$ of the unconstrained model.

The population item covariances and correlations for all model sizes are available in the online supplementary material.

**Table 2:** Study 2: Model size and number of groups configurations

*p*	*g*	$\text {df}_0$	$\text {df}_1$	*q*
5	2	14	10	4
	4	32	20	12
	8	68	40	28
10	2	79	70	9
	4	167	140	27
	8	343	280	63
20	2	359	340	19
	4	737	680	57
	8	1493	1360	133

*Note.* *p* = number of observed variables. *g* = number of groups. $q=\text {df}_0-\text {df}_1$. $\text {df}_0$ = degrees of freedom in the constrained model. $\text {df}_1$ = degrees of freedom in the unconstrained model

### Study 3: Power

In this study, we evaluated how well the best-performing test statistics in terms of type I error control were able to detect model misspecification. For each model specification employed in Study 1 and Study 2, we generate model misspecification by introducing a localized method effect. Specifically, we introduced an orthogonal method factor (*M*) that loaded exclusively on odd-numbered indicators of the first substantive factor ($F_1$). This method factor had unit variance and was uncorrelated with all substantive factors. To ensure the method factor represented a nuisance source of variance distinct from the substantive trait, the loadings of *M* were specified with alternating signs (e.g., $+\lambda , -\lambda , +\lambda ,\ldots$) on the subset of $F_1$ indicators. For $p=15, 30$, and 60, $\lambda$ was set to 0.78, 0.43, and 0.32, respectively. These values were chosen such that the rejection rate of RLS was approximately equal to 50% in each model, under normally distributed data of sample size $n=800$. The three data-generating models are fully listed in the online supplementary material. Due to limited computational resources, we did not include the $p=100$ case for power investigations. The total number of simulation conditions for the power of goodness-of-fit testing was therefore 63.

As an additional robustness check, we examined an alternative misspecification design in which, instead of an omitted method factor, misspecification was induced by including residual covariances and cross-loadings across the five factors. The corresponding data-generating models are provided in the online supplementary material.

For power under weak invariance testing, we employed data-generating non-invariant population models obtained by perturbing factor loadings across groups (as done, e.g., in Rutkowski & Svetina, 2017) of size *h*, where *h* was chosen to attain the required degree of non-invariance. The total number of simulation conditions for power of invariance testing is obtained by fully crossing the three sample sizes, three dimensions, three group sizes, and seven distributions, for a total of 189 conditions. All nine data-generating models are listed in the online supplementary material.

### Data generation

All analyses were conducted in the R environment (R Core Team, 2025). Model estimation was conducted using the package lavaan (Rosseel, 2012), which was also used to generate samples from the normal and VM distributions. Sample generation from the IG and PL distributions was conducted using the package covsim (Grønneberg et al., 2022). In each simulation condition, 2000 replicated datasets were created. Goodness-of-fit *p* values were calculated with the semTests package (Moss, 2024), with the exception of the nested model test $\text {SB}_\text {2001}$, which was calculated using lavaan.

Because of the large number of simulation conditions, many of which were high-dimensional, the simulation work was performed on resources provided by Sigma2 – the National Infrastructure for High-Performance Computing and Data Storage in Norway.

### Evaluation criteria

In each of the simulation conditions, we calculated the rejection rate (RR) associated with each test as the percentage of *p* values across the 2000 replications that were below the 5% level of significance. A complete list of these rejection rates in each condition, as well as plots, is reported in the online supplementary material. In the main text, we will mostly focus on aggregated results. To summarize these aggregated results, we follow Foldnes et al. (2025) and evaluate test procedure performance using three criteria.

The root-mean-square error (RMSE) criterion is given by $\text {RMSE}= \sqrt{\sum _c (RR_c - 5)^2/C}$, where *C* denotes the number of conditions. The mean absolute deviation is given by $\text {MAD}=\sum _c(|RR_c-5|)/C$. Both RMSE and MAD measure the distance from the nominal 5% rejection rate; so smaller values reflect better test performance. The interpretation of RMSE is the Euclidean distance between the rejection rates and a vector constantly equal to the nominal rejection rate. MAD is instead the average absolute distance between the individual rejection rates and the nominal rejection rate. While we will order tests relative to their RMSE performance, MAD has the advantage of having a more direct interpretation compared to the non-linear RMSE: If MAD is 0.1, then the average absolute deviation between the rejection rate and the nominal rate is simply $0.1 \%$.

A third criterion was proposed by Bradley (1978), namely the percentage of acceptable rejection rates (ARR), defined by the proportion of RRs between 2.5% and 7.5%. As a supplement to the ARR, we also report the proportion of RRs below 2.5% (B2.5) and the proportion of RRs above 7.5% (A7.5). All else equal, and under correct model specification, it is preferable that B2.5 > A7.5, in line with the requirement that the type I error rate should not exceed the nominal significance level. For example, Lehmann and Romano (2005, p. 57, Eq (3.2)) base the general theory of non-asymptotic hypothesis testing on the requirement that the type I error rate should not exceed the nominal significance level, and then aim to maximize power as a secondary requirement.

All of the evaluation criteria discussed here are subject to Monte Carlo sampling variability. In each condition, we used 2000 replications to reduce this variability. We have not quantified the variability, and limit ourselves to reporting the estimates without standard errors.

## Results

Full lists and visualizations of all test rejection rates in all conditions are available in the supplementary material.

**Fig. 1:**
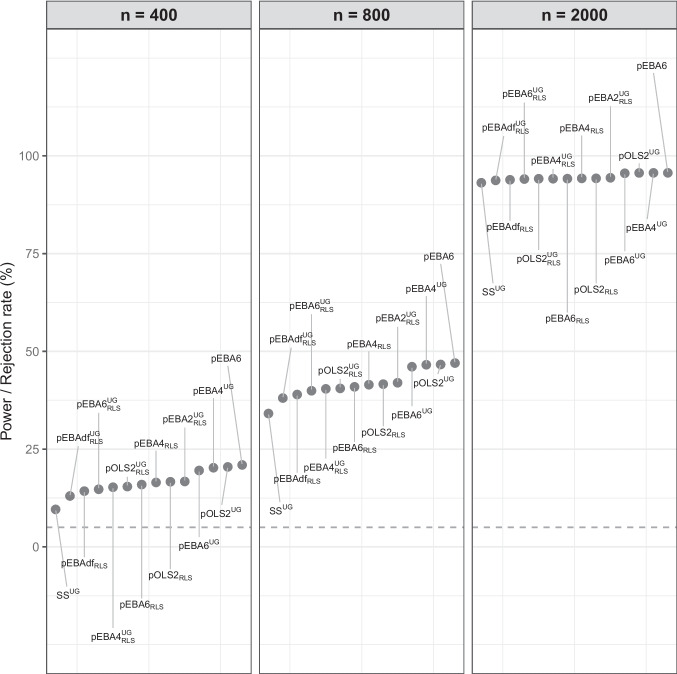
Study 3, goodness-of-fit testing for dimension 15. The panels display the rejection rates (power) for sample sizes $n = 400$, 800, and 2000. Within each panel, methods are sorted by their rejection rate

In the following, we report test performance aggregated over particularly interesting substrata of these conditions. The unaggregated tables in the supplementary material show mostly the same patterns as we report in the following. A visualization of these tables is given in *Study1_Unaggregated_plots.pdf* for Study 1, *Study2_Unaggregated_plots.pdf* for Study 2, and Fig. 1 for Study 3. From these figures, we see that the unaggregated results follow much the same patterns as what we describe in the following. Additional aggregated performance results are given in the online supplementary files *S1RMSE.pdf* and *S2RMSE.pdf*.

### Study 1

We first include only conditions with an acceptable sample size, i.e., where $n/q \ge 10$, in our analyses.

### Normal data with acceptable sample sizes

In Table 3, we list test performance according to RMSE for the six conditions where $n/q \ge 10$ and the data are normal. The normal-theory methods RLS and ML have a striking difference in performance, with the former being the overall winner, and the latter almost being the worst performer. The poor performance of $T_\text {ML}$ is carried over to the robustified tests, with the top 13 tests all being based on $T_\text {RLS}$. The superiority of RLS reported here is in accordance with previous studies (Foldnes et al., 2025; Hayakawa, 2019). Furthermore, as reported in the supplementary material, RLS remains the best performer in the six conditions where $n/q < 10$, with an RMSE of 0.63 and an ARR of $100\%$.

**Table 3:** Study 1. Normal data, aggregated over six conditions with acceptable sample sizes

Upper half (by RMSE rank)						Lower half (by RMSE rank)					
Method	RMSE	MAD	B2.5	ARR	A7.5	Method	RMSE	MAD	B2.5	ARR	A7.5
$\text {RLS}$	0.34	0.25	0.0	100.0	0.0	$\text {SF}$	1.29	1.05	0.0	100.0	0.0
$\text {SB}^\text {UG}_\text {RLS}$	0.38	0.33	0.0	100.0	0.0	$\text {pEBAdf}$	1.37	1.05	0.0	83.3	16.7
$\text {pEBA}2_\text {RLS}$	0.42	0.30	0.0	100.0	0.0	$\text {SS}^\text {UG}$	1.38	1.12	0.0	100.0	0.0
$\text {SB}_\text {RLS}$	0.42	0.35	0.0	100.0	0.0	$\text {pEBA}6$	1.46	1.12	0.0	83.3	16.7
$\text {pOLS}2_\text {RLS}$	0.64	0.51	0.0	100.0	0.0	$\text {pEBA}2^\text {UG}$	1.48	1.01	0.0	83.3	16.7
$\text {pEBA}4_\text {RLS}$	0.64	0.52	0.0	100.0	0.0	$\text {ALL}^\text {UG}$	1.49	1.25	0.0	100.0	0.0
$\text {pEBA}6_\text {RLS}$	0.73	0.58	0.0	100.0	0.0	$\text {SF}^\text {UG}$	1.49	1.25	0.0	100.0	0.0
$\text {pEBAdf}_\text {RLS}$	0.75	0.60	0.0	100.0	0.0	$\text {pEBA}4$	1.58	1.20	0.0	83.3	16.7
$\text {pEBA}2^\text {UG}_\text {RLS}$	0.79	0.65	0.0	100.0	0.0	$\text {pOLS}2$	1.60	1.21	0.0	83.3	16.7
$\text {pEBA}4^\text {UG}_\text {RLS}$	0.91	0.77	0.0	100.0	0.0	$\text {pEBA}2$	1.90	1.41	0.0	83.3	16.7
$\text {pOLS}2^\text {UG}_\text {RLS}$	0.91	0.77	0.0	100.0	0.0	$\text {SS}_\text {RLS}$	2.25	1.91	33.3	66.7	0.0
$\text {pEBA}6^\text {UG}_\text {RLS}$	0.93	0.78	0.0	100.0	0.0	$\text {ALL}_\text {RLS}$	2.33	2.01	33.3	66.7	0.0
$\text {pEBAdf}^\text {UG}_\text {RLS}$	1.00	0.84	0.0	100.0	0.0	$\text {SF}_\text {RLS}$	2.33	2.01	33.3	66.7	0.0
$\text {pEBAdf}^\text {UG}$	1.04	0.74	0.0	100.0	0.0	$\text {SB}^\text {UG}$	2.34	1.66	0.0	66.7	33.3
$\text {pEBA}6^\text {UG}$	1.07	0.76	0.0	100.0	0.0	$\text {SS}^\text {UG}_\text {RLS}$	2.42	2.11	33.3	66.7	0.0
$\text {SS}$	1.09	0.88	0.0	100.0	0.0	$\text {ALL}^\text {UG}_\text {RLS}$	2.49	2.21	33.3	66.7	0.0
$\text {pEBA}4^\text {UG}$	1.12	0.78	0.0	100.0	0.0	$\text {SF}^\text {UG}_\text {RLS}$	2.49	2.21	33.3	66.7	0.0
$\text {pOLS}2^\text {UG}$	1.14	0.79	0.0	83.3	16.7	$\text {ML}$	2.78	1.99	0.0	66.7	33.3
$\text {ALL}$	1.29	1.05	0.0	100.0	0.0	$\text {SB}$	2.84	2.09	0.0	66.7	33.3

*Note.* RMSE = root mean squared error. MAD = mean absolute percentage points deviation from 5%. B2.5 = proportion of conditions with a rejection rate $<2.5\%$. ARR = proportion of conditions with a rejection rate between $2.5\%$ and $7.5\%$. A7.5 = proportion of conditions with a rejection rate $>7.5\%$

**Table 4:** Study 1. Non-normal data aggregated over 36 conditions with acceptable sample sizes

Upper half (by RMSE rank)						Lower half (by RMSE rank)					
Method	RMSE	MAD	B2.5	ARR	A7.5	Method	RMSE	MAD	B2.5	ARR	A7.5
$\text {pEBA}6^\text {UG}$	1.03	0.75	0.0	97.2	2.8	$\text {pEBAdf}^\text {UG}_\text {RLS}$	2.24	1.98	38.9	61.1	0.0
$\text {pEBA}4_\text {RLS}$	1.05	0.87	0.0	97.2	2.8	$\text {SB}_\text {RLS}$	2.42	1.66	0.0	75.0	25.0
$\text {pOLS}2_\text {RLS}$	1.06	0.89	0.0	97.2	2.8	$\text {pEBA}2$	2.63	1.88	0.0	69.4	30.6
$\text {pEBA}6$	1.12	0.77	0.0	94.4	5.6	$\text {SB}^\text {UG}$	3.61	2.70	0.0	58.3	41.7
$\text {pEBA}6_\text {RLS}$	1.14	0.97	0.0	100.0	0.0	$\text {SS}$	3.68	3.48	77.8	22.2	0.0
$\text {pEBA}2^\text {UG}_\text {RLS}$	1.20	0.96	0.0	97.2	2.8	$\text {SS}^\text {UG}$	3.74	3.56	80.6	19.4	0.0
$\text {pEBA}4^\text {UG}_\text {RLS}$	1.23	1.04	0.0	100.0	0.0	$\text {ALL}$	3.82	3.64	80.6	19.4	0.0
$\text {pEBA}4^\text {UG}$	1.23	0.85	0.0	94.4	5.6	$\text {SF}$	3.83	3.66	80.6	19.4	0.0
$\text {pOLS}2^\text {UG}$	1.24	0.89	0.0	91.7	8.3	$\text {SS}_\text {RLS}$	3.87	3.71	83.3	16.7	0.0
$\text {pOLS}2^\text {UG}_\text {RLS}$	1.26	1.09	0.0	97.2	2.8	$\text {ALL}^\text {UG}$	3.90	3.74	83.3	16.7	0.0
$\text {pEBA}2_\text {RLS}$	1.34	0.94	0.0	91.7	8.3	$\text {SF}^\text {UG}$	3.90	3.75	83.3	16.7	0.0
$\text {pEBAdf}$	1.35	1.03	5.6	94.4	0.0	$\text {SS}^\text {UG}_\text {RLS}$	3.93	3.78	83.3	16.7	0.0
$\text {pEBA}6^\text {UG}_\text {RLS}$	1.38	1.20	5.6	94.4	0.0	$\text {ALL}_\text {RLS}$	3.99	3.84	86.1	13.9	0.0
$\text {pEBA}4$	1.45	1.00	0.0	91.7	8.3	$\text {SF}_\text {RLS}$	4.00	3.85	86.1	13.9	0.0
$\text {pOLS}2$	1.47	0.99	0.0	91.7	8.3	$\text {ALL}^\text {UG}_\text {RLS}$	4.05	3.92	86.1	13.9	0.0
$\text {pEBAdf}^\text {UG}$	1.55	1.26	13.9	86.1	0.0	$\text {SF}^\text {UG}_\text {RLS}$	4.05	3.92	86.1	13.9	0.0
$\text {SB}^\text {UG}_\text {RLS}$	2.01	1.38	0.0	75.0	25.0	$\text {SB}$	4.15	3.21	0.0	55.6	44.4
$\text {pEBAdf}_\text {RLS}$	2.03	1.77	30.6	69.4	0.0	$\text {RLS}$	52.85	39.44	0.0	5.6	94.4
$\text {pEBA}2^\text {UG}$	2.17	1.47	0.0	75.0	25.0	$\text {ML}$	54.38	41.65	0.0	2.8	97.2

*Note.* RMSE = root mean squared error. MAD = mean absolute percentage points deviation from 5%. B2.5 = proportion of conditions with a rejection rate $<2.5$%. ARR = proportion of conditions with a rejection rate between 2.5% and 7.5%. A7.5 = proportion of conditions with a rejection rate $>7.5$%

**Table 5:** Study 1. Rejection rates for each distributional condition in the $p=100$ case with $n=2000$

First half (alphabetical)								Last half (alphabetical)							
Method	N	VM1	PL1	IG1	VM2	PL2	IG2	Method	N	VM1	PL1	IG1	VM2	PL2	IG2
ALL	1.1	0.0	0.1	0.1	0.0	0.0	0.0	$\text {pEBA6}^\text {UG}_\text {RLS}$	1.4	1.3	1.8	1.5	3.5	2.7	3.7
$\text {ALL}_\text {RLS}$	0.1	0.0	0.0	0.0	0.0	0.0	0.0	$\text {pOLS2}$	12.0	4.1	4.2	5.3	1.0	1.1	6.2
$\text {ALL}^\text {UG}$	1.0	0.0	0.1	0.1	0.0	0.0	0.0	$\text {pOLS2}_\text {RLS}$	1.7	0.6	0.8	0.7	1.2	0.8	1.3
$\text {ALL}^\text {UG}_\text {RLS}$	0.1	0.0	0.0	0.0	0.0	0.0	0.0	$\text {pOLS2}^\text {UG}$	10.6	3.0	3.6	4.6	0.8	1.0	5.6
$\text {pEBAdf}$	12.0	3.3	4.9	6.0	0.3	0.7	3.0	$\text {pOLS2}^\text {UG}_\text {RLS}$	1.3	0.5	0.7	0.5	1.0	0.7	1.1
$\text {pEBAdf}_\text {RLS}$	1.6	0.5	1.0	0.9	0.1	0.4	0.5	$\text {SB}$	23.8	19.5	21.9	23.6	16.5	19.4	28.1
$\text {pEBAdf}^\text {UG}$	10.6	2.5	4.1	5.0	0.2	0.6	2.7	$\text {SB}_\text {RLS}$	5.5	6.5	6.7	5.6	13.0	11.4	9.9
$\text {pEBAdf}^\text {UG}_\text {RLS}$	1.3	0.5	0.8	0.6	0.1	0.3	0.5	$\text {SB}^\text {UG}$	21.9	17.5	19.8	21.9	14.6	17.1	25.4
$\text {pEBA2}$	18.2	14.5	16.9	18.5	12.8	14.8	21.9	$\text {SB}^\text {UG}_\text {RLS}$	4.4	5.7	6.0	5.0	11.5	10.1	9.1
$\text {pEBA2}_\text {RLS}$	3.6	4.5	4.9	4.0	10.4	8.7	7.5	$\text {SF}$	1.1	0.0	0.1	0.1	0.0	0.0	0.0
$\text {pEBA2}^\text {UG}$	16.1	13.2	15.0	16.7	10.9	13.0	20.0	$\text {SF}_\text {RLS}$	0.1	0.0	0.0	0.0	0.0	0.0	0.0
$\text {pEBA2}^\text {UG}_\text {RLS}$	2.7	3.7	4.2	3.3	9.0	7.9	6.8	$\text {SF}^\text {UG}$	1.0	0.0	0.1	0.1	0.0	0.0	0.0
$\text {pEBA}4$	13.8	9.8	10.6	12.7	7.3	9.1	15.5	$\text {SF}^\text {UG}_\text {RLS}$	0.1	0.0	0.0	0.0	0.0	0.0	0.0
$\text {pEBA}4_\text {RLS}$	2.1	2.4	3.2	2.5	6.0	4.9	4.9	$\text {SS}$	1.2	0.0	0.1	0.1	0.0	0.0	0.0
$\text {pEBA}4^\text {UG}$	12.3	8.9	9.5	11.0	6.6	8.0	14.1	$\text {SS}_\text {RLS}$	0.2	0.0	0.0	0.0	0.0	0.0	0.0
$\text {pEBA}4^\text {UG}_\text {RLS}$	1.7	1.9	2.7	2.1	5.2	4.3	4.5	$\text {SS}^\text {UG}$	1.1	0.0	0.1	0.1	0.0	0.0	0.0
$\text {pEBA6}$	12.7	8.1	8.5	10.3	5.3	6.2	12.5	$\text {SS}^\text {UG}_\text {RLS}$	0.1	0.0	0.0	0.0	0.0	0.0	0.0
$\text {pEBA6}_\text {RLS}$	2.0	1.7	2.0	1.9	4.1	3.6	3.9	ML	23.2	100.0	100.0	60.6	100.0	100.0	92.5
$\text {pEBA6}^\text {UG}$	11.6	7.2	7.2	8.8	4.2	5.6	11.2	RLS	5.0	100.0	100.0	30.3	100.0	100.0	79.9

*Note.* RMSE = root mean squared error. MAD = mean absolute percentage points deviation from 5%. B2.5 = proportion of conditions with a rejection rate $<2.5\%$. ARR = proportion of conditions with a rejection rate between $2.5\%$ and $7.5\%$. A7.5 = proportion of conditions with a rejection rate $>7.5\%$. Distribution: N = normal, VM = Vale-Maurelli, PL = Piecewise linear, IG = Independent generator. Numbers after the distribution abbreviation indicate (1): moderate skewness and kurtosis, and (2) severe skewness and kurtosis

### Non-normal data with acceptable sample sizes

There were 36 conditions with $n/q \ge 10$ under non-normal distributions. In Table 4, we can see that the best-performing test in terms of RMSE is $\text {pEBA}6^\text {UG}$, closely followed by $\text {pEBA}4_\text {RLS}$. As expected, the normal-theory tests ML and RLS have extremely poor performance under non-normality. The SB test based on $T_\text {ML}$ and the biased $\Gamma$ estimator is a default in popular software packages lavaan and Mplus (Muthén & Muthén, 2017). Surprisingly, this test has the poorest performance among all robustified tests, with an RMSE four times as high as that of the best performing tests, which all belong to the pEBA/pOLS class of procedures. Echoing the findings of Foldnes et al. (2025), the best version of SB, $\text {SB}^\text {UG}_\text {RLS}$, is based on $T_\text {RLS}$ and the unbiased $\Gamma$ estimator.

As reported in the supplementary material, $\text {pEBA}6^\text {UG}$ performs poorly in the 36 conditions with $n/q < 10$, with an RMSE of 38.7 and an ARR of 33%. In contrast, $\text {pEBA}4_\text {RLS}$ has a much better performance in these sub-optimal conditions, with an RMSE of 4.9 and an ARR of 75%.

### The high-dimensional case

Here we consider the special case of $p=100$ with $n=2000$ observations, which is close to an acceptable sample size, with $n/q =9.5$. For smaller sample sizes, the test performance generally deteriorates, with similar patterns as we here report for the $n=2000$ case.

Table 5 lists the rejection rates in each distributional condition for the 100-dimensional model with $n=2000$, while Table 6 ranks the tests according to RMSE calculated across these seven distributional conditions.

**Table 6:** Study 1. The $p=100$, $n=2000$ case aggregated over seven distributional conditions

Ranks 1–19 (by RMSE)						Ranks 20–38 (by RMSE)					
Method	RMSE	MAD	B2.5	ARR	A7.5	Method	RMSE	MAD	B2.5	ARR	A7.5
$\text {pEBA}4_\text {RLS}$	1.93	1.60	42.9	57.1	0.0	$\text {SF}$	4.84	4.83	100.0	0.0	0.0
$\text {pEBA}4^\text {UG}_\text {RLS}$	2.25	1.88	42.9	57.1	0.0	$\text {SS}^\text {UG}$	4.84	4.83	100.0	0.0	0.0
$\text {pEBA}2^\text {UG}_\text {RLS}$	2.33	2.12	0.0	71.4	28.6	$\text {ALL}^\text {UG}$	4.85	4.84	100.0	0.0	0.0
$\text {pEBA}6_\text {RLS}$	2.50	2.29	57.1	42.9	0.0	$\text {SF}^\text {UG}$	4.85	4.84	100.0	0.0	0.0
$\text {pEBA}2_\text {RLS}$	2.74	2.09	0.0	57.1	42.9	$\text {pEBA}6$	4.88	4.07	0.0	28.6	71.4
$\text {pEBA}6^\text {UG}_\text {RLS}$	2.90	2.75	57.1	42.9	0.0	$\text {SS}_\text {RLS}$	4.98	4.98	100.0	0.0	0.0
$\text {pOLS}2^\text {UG}$	3.20	2.60	28.6	57.1	14.3	$\text {ALL}^\text {UG}_\text {RLS}$	4.99	4.99	100.0	0.0	0.0
$\text {pOLS}2$	3.46	2.59	28.6	57.1	14.3	$\text {ALL}_\text {RLS}$	4.99	4.99	100.0	0.0	0.0
$\text {SB}^\text {UG}_\text {RLS}$	3.51	2.56	0.0	57.1	42.9	$\text {SF}^\text {UG}_\text {RLS}$	4.99	4.99	100.0	0.0	0.0
$\text {pEBAdf}^\text {UG}$	3.53	2.95	42.9	42.9	14.3	$\text {SF}_\text {RLS}$	4.99	4.99	100.0	0.0	0.0
$\text {pEBAdf}$	3.75	2.99	28.6	57.1	14.3	$\text {SS}^\text {UG}_\text {RLS}$	4.99	4.99	100.0	0.0	0.0
$\text {pEBA}6^\text {UG}$	3.90	3.18	0.0	57.1	42.9	$\text {pEBA}4^\text {UG}$	5.58	5.04	0.0	14.3	85.7
$\text {pOLS}2_\text {RLS}$	4.01	4.00	100.0	0.0	0.0	$\text {pEBA}4$	6.80	6.25	0.0	14.3	85.7
$\text {pOLS}2^\text {UG}_\text {RLS}$	4.20	4.19	100.0	0.0	0.0	$\text {pEBA}2^\text {UG}$	10.37	9.99	0.0	0.0	100.0
$\text {pEBAdf}_\text {RLS}$	4.32	4.30	100.0	0.0	0.0	$\text {pEBA}2$	12.12	11.78	0.0	0.0	100.0
$\text {SB}_\text {RLS}$	4.37	3.37	0.0	57.1	42.9	$\text {SB}^\text {UG}$	15.11	14.72	0.0	0.0	100.0
$\text {pEBAdf}^\text {UG}_\text {RLS}$	4.46	4.44	100.0	0.0	0.0	$\text {SB}$	17.20	16.84	0.0	0.0	100.0
$\text {SS}$	4.83	4.81	100.0	0.0	0.0	$\text {RLS}$	77.78	68.60	0.0	14.3	85.7
$\text {ALL}$	4.84	4.83	100.0	0.0	0.0	$\text {ML}$	82.10	77.33	0.0	0.0	100.0

*Note.* RMSE = root mean squared error. MAD = mean absolute percentage points deviation from 5%. B2.5 = proportion of conditions with a rejection rate $<2.5\%$. ARR = proportion of conditions with a rejection rate between $2.5\%$ and $7.5\%$. A7.5 = proportion of conditions with a rejection rate $>7.5\%$

**Table 7:** Study 1. Ten best methods, according to RMSE, for the 42 conditions where $n/q>10$

Method	RMSE	MAD	B2.5	ARR	A7.5
$\text {pEBA}4_\text {RLS}$	1.00	0.82	0.0	97.6	2.4
$\text {pOLS}2_\text {RLS}$	1.01	0.84	0.0	97.6	2.4
$\text {pEBA}6^\text {UG}$	1.03	0.75	0.0	97.6	2.4
$\text {pEBA}6_\text {RLS}$	1.09	0.92	0.0	100.0	0.0
$\text {pEBA}2^\text {UG}_\text {RLS}$	1.15	0.92	0.0	97.6	2.4
$\text {pEBA}6$	1.17	0.82	0.0	92.9	7.1
$\text {pEBA}4^\text {UG}_\text {RLS}$	1.19	1.01	0.0	100.0	0.0
$\text {pEBA}4^\text {UG}$	1.21	0.84	0.0	95.2	4.8
$\text {pOLS}2^\text {UG}_\text {RLS}$	1.22	1.05	0.0	97.6	2.4
$\text {pOLS}2^\text {UG}$	1.23	0.87	0.0	90.5	9.5

*Note.* RMSE = root mean squared error. MAD = mean absolute percentage points deviation from 5%. B2.5 = proportion of conditions with a rejection rate $<2.5\%$. ARR = proportion of conditions with a rejection rate between $2.5\%$ and $7.5\%$. A7.5 = proportion of conditions with a rejection rate $>7.5\%$

**Table 8:** Study 1. Ten best methods, according to RMSE, for the 42 conditions where $n/q \le 10$

Method	RMSE	MAD	B2.5	ARR	A7.5
$\text {pEBA}6^\text {UG}_\text {RLS}$	3.16	2.89	57.1	40.5	2.4
$\text {pEBA}4^\text {UG}_\text {RLS}$	3.19	2.53	45.2	47.6	7.1
$\text {pEBA}6_\text {RLS}$	3.50	2.63	40.5	52.4	7.1
$\text {pOLS}2_\text {RLS}$	3.68	3.42	73.8	26.2	0.0
$\text {pOLS}2^\text {UG}_\text {RLS}$	3.97	3.79	83.3	16.7	0.0
$\text {pEBAdf}_\text {RLS}$	4.41	4.33	97.6	2.4	0.0
$\text {pEBA}2^\text {UG}_\text {RLS}$	4.54	2.90	4.8	69.0	26.2
$\text {pEBAdf}^\text {UG}_\text {RLS}$	4.57	4.52	97.6	2.4	0.0
$\text {pEBA}4_\text {RLS}$	4.66	2.75	11.9	71.4	16.7
$\text {SS}^\text {UG}$	4.79	4.77	97.6	0.0	2.4

*Note.* RMSE = root mean squared error. MAD = mean absolute percentage points deviation from 5%. B2.5 = proportion of conditions with a rejection rate $<2.5\%$. ARR = proportion of conditions with a rejection rate between $2.5\%$ and $7.5\%$. A7.5 = proportion of conditions with a rejection rate $>7.5\%$

**Table 9:** Study 2. Aggregated over all 189 conditions

Upper half (by RMSE rank)						Lower half (by RMSE rank)					
Method	RMSE	MAD	B2.5	ARR	A7.5	Method	RMSE	MAD	B2.5	ARR	A7.5
$\text {pEBAdf}^\text {UG}$	0.946	0.692	0.0	98.4	1.6	$\text {pEBA}2^\text {UG}$	2.121	1.293	0.0	85.7	14.3
$\text {pEBAdf}$	1.172	0.843	0.0	96.3	3.7	$\text {SB}_\text {2001}$	2.131	1.566	22.8	76.7	0.5
$\text {SS}_\text {RLS}$	1.418	1.071	2.1	96.8	0.5	$\text {pEBAdf}^\text {UG}_\text {RLS}$	2.241	0.958	0.0	95.2	4.8
$\text {pEBA}6^\text {UG}$	1.443	0.912	0.0	91.0	9.0	$\text {pEBAdf}_\text {RLS}$	2.469	1.091	0.0	93.1	6.9
$\text {SS}$	1.482	1.147	10.1	87.8	0.0	$\text {pEBA}2$	2.470	1.549	0.0	82.0	18.0
$\text {SS}^\text {UG}_\text {RLS}$	1.489	1.179	4.8	92.6	0.5	$\text {SB}^\text {UG}$	2.811	1.784	0.0	77.2	22.8
$\text {SF}_\text {RLS}$	1.561	1.245	10.6	88.9	0.5	$\text {pEBA}6^\text {UG}_\text {RLS}$	3.051	1.320	0.0	87.3	12.7
$\text {ALL}_\text {RLS}$	1.580	1.270	10.6	88.4	0.5	$\text {SB}$	3.188	2.077	0.0	70.9	28.0
$\text {SS}^\text {UG}$	1.612	1.250	13.8	85.2	0.0	$\text {pEBA}4^\text {UG}_\text {RLS}$	3.276	1.437	0.0	85.2	14.3
$\text {pEBA}4^\text {UG}$	1.635	1.005	0.0	89.4	10.6	$\text {pEBA}6_\text {RLS}$	3.333	1.492	0.0	84.7	15.3
$\text {SF}^\text {UG}_\text {RLS}$	1.661	1.365	12.2	85.7	0.5	$\text {pOLS}2^\text {UG}_\text {RLS}$	3.337	1.466	0.0	85.2	14.8
$\text {ALL}^\text {UG}_\text {RLS}$	1.685	1.387	13.2	85.2	0.5	$\text {pEBA}4_\text {RLS}$	3.564	1.614	0.0	82.5	16.9
$\text {pOLS}2^\text {UG}$	1.697	1.034	0.0	89.4	10.6	$\text {pOLS}2_\text {RLS}$	3.656	1.654	0.0	82.0	18.0
$\text {pEBA}6$	1.704	1.100	0.0	88.9	11.1	$\text {pEBA}2^\text {UG}_\text {RLS}$	3.802	1.752	0.0	81.0	19.0
$\text {SF}$	1.716	1.330	17.5	82.0	0.0	$\text {pEBA}2_\text {RLS}$	4.114	1.963	0.0	78.8	20.6
$\text {ALL}$	1.727	1.344	18.0	82.0	0.0	$\text {SB}^\text {UG}_\text {RLS}$	4.418	2.203	0.0	75.7	24.3
$\text {SF}^\text {UG}$	1.834	1.441	21.2	78.3	0.0	$\text {SB}_\text {RLS}$	4.757	2.439	0.0	73.5	25.4
$\text {ALL}^\text {UG}$	1.848	1.458	21.2	78.3	0.0	$\text {RLS}$	75.201	67.147	0.0	14.3	85.7
$\text {pEBA}4$	1.931	1.218	0.0	87.3	12.7	$\text {ML}$	75.658	67.692	0.0	14.3	85.7
$\text {pOLS}2$	1.996	1.247	0.0	86.8	12.7						

*Note.* RMSE = root mean squared error. MAD = mean absolute percentage points deviation from 5%. B2.5 = proportion of conditions with a rejection rate $<2.5\%$. ARR = proportion of conditions with a rejection rate between $2.5\%$ and $7.5\%$. A7.5 = proportion of conditions with a rejection rate $>7.5\%$

Established methods (ML, RLS, SS, SB based on biased $\Gamma$ and $T_\text {ML}$) all have unacceptable rejection rates for every distribution, except for RLS in the normal case. Remarkably, as reported in the supplementary material, even under smaller sample sizes, RLS maintains a close-to-nominal rejection rate under normality (6.0% and 5.9% for $n=800$ and $n=400$, respectively). Under normality, Moshagen (2012) also studied the rejection rate of ML in the high dimensional case, and found a severe over-rejection of near $100 \%$, in line with our findings.

The conventional version of SB performs the worst of all robustified tests, according to RMSE. Among the modified versions of established tests, $\text {SB}^\text {UG}_\text {RLS}$ performs best, echoing the findings reported above for conditions with acceptable sample sizes. Still, many versions of the new penalized procedures outperform all established tests, with $\text {pEBA}4_\text {RLS}$ attaining a MAD of 1.6%, compared to a MAD of 2.6% for $\text {SB}^\text {UG}_\text {RLS}$. Also, $\text {SB}^\text {UG}_\text {RLS}$ has a large proportion of conditions with RR > 7.5%, comparing unfavorably to $\text {pEBA}4_\text {RLS}$ also on this criterion.

### Overall performance

In Table 7, we tabulate the ten best methods across the 42 simulation conditions where sample size is acceptable, i.e., where $n/q>10$. All of these methods are penalized test procedures, and perform well, each with acceptable rejection rates in more than 90% of the conditions. Again, $\text {pEBA}4_\text {RLS}$ is the best performer, echoing the findings in the previous section.

Table 8 lists the top ten tests for the suboptimal 42 conditions with $n/q < 10$. As expected, the RMSE/MAD criteria are elevated relative to the findings in Table 7. Here, $\text {pEBA}4_\text {RLS}$ is ranked 9th according to RMSE, 3rd according to MAD, and 1st according to ARR.

### Study 2

Table 9 lists test performance as calculated across all 189 conditions of sample sizes, model sizes, group sizes, and distributions. As documented in the supplementary material, test performance is overall improved when calculating $U_D$ from Satorra (2000), compared to using Satorra and Bentler (2001). Therefore, with the exception of $\text {SB}_\text {2001}$, which is included due to its extensive use in empirical work, being the default test in lavaan, we here report only findings based on the Satorra (2000) method. The unaggregated results are available in the supplementary material.

The overall winner is $\text {pEBAdf}^\text {UG}$, with RMSE = 0.946, MAD = 0.692, and ARR = 98.4%. Remarkably, across the 189 conditions, only three conditions had unacceptable rejection rates. These three conditions involved eight groups and sample sizes of 400: For dimension 10 and distribution PL2, the rejection rate was 7.6%. For dimension 20 and distributions VM2 and PL2, the rejection rate was 8.1% and 9%, respectively.

Given this generally satisfactory performance across all conditions of sample size, model size, number of groups, and distributions, we here report mainly on aggregated performance, referring interested readers to the supplementary material for unaggregated results.

Among the established methods (ML, RLS, SS, SB based on biased $\Gamma$ and $T_\text {ML}$), SS performs well and is ranked 5th (RMSE = 1.482), while $\text {SB}_\text {2001}$ has a mediocre performance (RMSE = 2.131). The version of SS based on RLS has an even better performance (RMSE = 1.418), and is ranked 3rd. Moreover, $\text {SS}_\text {RLS}$ very seldom overrejects (0.5% A7.5 in Table 9), which is preferable relative to $\text {pEBAdf}^\text {UG}$ (1.6% A7.5).

Under normality, as reported in the supplementary material, all test procedures performed well, all reaching an ARR of 100%, with $\text {ALL}^\text {UG}$ performing the best (RMSE = 0.444, MAD = 0.324) across the 27 normal conditions. The overall winner $\text {pEBAdf}^\text {UG}$ is ranked 4th under normality, with a comparable RMSE of 0.472. Although not top-10 contenders, RLS and ML perform well under normality with RMSE of 0.489 and 0.607, respectively.

**Table 10:** Study 3. CFA goodness-of-fit testing. Power by method across dimensions *p*= 15, 30, 60, and combined, for conditions with adequate sample size. The highest power in each dimension is boldfaced

Method	Power (dim=15)	Power (dim=30)	Power (dim=60)	Power overall
pEBA2$^\text {UG}_\text {RLS}$	51.0	**65.2**	**88.6**	**68.3**
pEBA4$^\text {UG}$	54.2	64.5	81.8	66.8
pEBA4$_\text {RLS}$	50.7	64.2	86.7	67.2
pEBA4$^\text {UG}_\text {RLS}$	50.0	63.1	85.9	66.3
pEBA6	**54.5**	64.6	81.0	66.7
pEBA6$^\text {UG}$	53.7	63.4	80.1	65.7
pEBA6$_\text {RLS}$	50.4	63.1	85.3	66.2
pEBA6$^\text {UG}_\text {RLS}$	49.6	62.1	84.4	65.3
pEBAdf$_\text {RLS}$	49.0	59.2	77.8	62.0
pEBAdf$^\text {UG}_\text {RLS}$	48.3	58.2	76.9	61.1
pOLS2$^\text {UG}$	54.3	64.8	80.3	66.4
pOLS2$_\text {RLS}$	50.9	64.4	85.3	66.8
pOLS2$^\text {UG}_\text {RLS}$	50.0	63.3	84.5	65.9
SS$^\text {UG}$	45.6	44.7	45.0	45.1

**Table 11:** Study 3. CFA goodness-of-fit testing. Power by method across dimensions *p*= 30, 60, and combined, for conditions with inadequate sample size. The highest power in each dimension is boldfaced

Method	Power (dim=30)	Power (dim=60)	Power Overall
pEBA2$^\text {UG}_\text {RLS}$	17.9	28.2	23.0
pEBA4$^\text {UG}$	21.9	**46.5**	34.2
pEBA4$_\text {RLS}$	16.7	24.9	20.8
pEBA4$^\text {UG}_\text {RLS}$	14.3	20.7	17.5
pEBA6	**22.7**	46.4	**34.5**
pEBA6$^\text {UG}$	19.8	39.9	29.9
pEBA6$_\text {RLS}$	15.0	20.5	17.7
pEBA6$^\text {UG}_\text {RLS}$	12.6	17.1	14.8
pEBAdf$_\text {RLS}$	9.5	9.3	9.4
pEBAdf$^\text {UG}_\text {RLS}$	8.0	7.7	7.9
pOLS2$^\text {UG}$	21.4	31.9	26.6
pOLS2$_\text {RLS}$	16.3	16.1	16.2
pOLS2$^\text {UG}_\text {RLS}$	14.0	13.3	13.6
SS$^\text {UG}$	2.9	2.4	2.7

### Study 3

#### Power of the goodness-of-fit test

We included in our evaluation the union of the top ten best performing tests in Tables 7 and 8. This resulted in 14 test statistics. In Fig. 1, we plot the rejection rates for the model with dimension 15, where we aggregate over all seven distributions. Expectedly, power increases with sample size. At $n=400$, power is fairly low, even though the sample size is adequate ($n/q \ge 10$). At all sample sizes, $SS_\text {UG}$ has the lowest power of all methods, with the pEBAdf variants also having poor power. pEBA6 is the most powerful test statistic at all sample sizes.

Let us now also aggregate over dimensions and consider the conditions with an adequate sample size $n/q \ge 10$. Table 10 lists the power attained for each statistic, aggregated across all distributions. The last column contains the overall power across all 42 $n/q \ge 10$ conditions. Power is seen to increase with increasing dimension. All the penalized eigenvalue methods markedly and consistently outperform the only included established method, $\text {SS}^\text {UG}$. This is unsurprising, given the strong tendency of SS to under-reject a true model (Foldnes & Olsson, 2015), also documented for all SS variants in the file *S1RMSE.pdf* in the online supplementary material. Among the penalized eigenvalue methods, the overall winner is pEBA2$^\text {UG}_\text {RLS}$ (68.3%), with all of the pEBA4 and pEBA6 variants as close runners-up. The best performing test in Study 1, $\text {pEBA}4_\text {RLS}$ has a slightly lower overall power (67.2%) than pEBA2$^\text {UG}_\text {RLS}$. However, prioritizing type I error over power implies that $\text {pEBA}4_\text {RLS}$ remains the recommended procedure for adequate sample sizes.

**Table 12:** Study 3. Weak invariance testing. Power by method across dimensions $p=5, 10, 20$, and overall. (There are 63 conditions for each dimension, and 189 conditions overall)

Method	Power (dim=5)	Power (dim=10)	Power (dim=20)	Overall
$\text {ALL}_\text {RLS}$	10.2	24.1	57.2	30.5
$\text {pEBA}4^\text {UG}$	11.7	**29.1**	**66.0**	**35.6**
$\text {pEBA}6^\text {UG}$	11.6	28.9	65.6	35.4
$\text {pEBAdf}$	**11.8**	28.7	65.0	35.2
$\text {pEBAdf}^\text {UG}$	11.5	28.2	64.3	34.7
$\text {SF}_\text {RLS}$	10.2	24.2	57.3	30.6
$\text {SS}$	10.5	25.0	58.5	31.3
$\text {SS}^\text {UG}$	10.3	24.6	57.8	30.9
$\text {SS}^\text {UG}_\text {RLS}$	10.2	24.4	57.9	30.8
$\text {SS}_\text {RLS}$	10.4	24.8	58.5	31.3

Next, we consider the 42 conditions with inadequate sample size $n/q < 10$, see Table 11. Again, $\text {SS}^\text {UG}$ stands out with overall very poor power (2.7%). The five methods $\text {pEBA}2^\text {UG}_\text {RLS}$ (23.0%), $\text {pEBA}4^\text {UG}$ (34.2%), $\text {pEBA}6$ (34.5%), $\text {pEBA}6^\text {UG}$ (29.9%), and $\text {pOLS}2^\text {UG}$ (26.6%) have markedly higher power than $\text {pEBA}4_\text {RLS}$ (20.8%).

This is unsurprising, given the strong tendency of these four tests to overreject correct models, as documented in the supplemental file *S1RMSE.pdf*. Given the importance of type I error rates at or below the nominal level, we retain $\text {pEBA}4_\text {RLS}$ as the recommended procedure for inadequate sample sizes.

Discarded from the above results due to poor type I error control, ML and RLS are nevertheless of special interest to applied researchers; we therefore report their power performance. Under adequate sample sizes ($n/q \ge 10$), baseline power was 56.1% (ML) and 52.6% (RLS) for $p=15$, 74.1% (ML) and 72.3% (RLS) for $p=30$, and 96.0% (ML) and 96.7% (RLS) for $p=60$. Under inadequate sample sizes ($n/q < 10$), baseline power was 61.2% (ML) and 54.0% (RLS) for $p=30$ and 86.0% (ML) and 70.4% (RLS) for $p=60$.

Results for the alternative robustness condition involving residual covariances and cross-loadings are reported in the online supplementary material. The qualitative pattern of findings was similar to that reported above, suggesting that the conclusions are robust across alternative model misspecifications.

#### Power of weak invariance testing

In Table 12 we present aggregated power for each dimension, as well as the overall power across all 189 conditions. In general, power increases with increasing dimension. The overall winner is $\text {pEBA}4^\text {UG}$ (35.6%), with $\text {pEBA}6^\text {UG}$ (35.4%), $\text {pEBAdf}$ (35.2%), and $\text {pEBAdf}^\text {UG}$ (34.7%) as runners-up. Among these, again giving priority to type I error control, we retain $\text {pEBAdf}^\text {UG}$ as the recommended test.

In Study 2, $\text {SS}_\text {RLS}$ was ranked 3rd and, in its favor, had a slightly lower tendency to overreject compared to $\text {pEBAdf}^\text {UG}$. $\text {SS}_\text {RLS}$ has a power of 31.3%, in disfavor relative to $\text {pEBAdf}^\text {UG}$. This leads us to retain $\text {pEBAdf}^\text {UG}$ as the recommended method, while recognizing that $\text {SS}_\text {RLS}$ is also a viable option. The same argument may be applied to the other SS versions and to $\text {ALL}_\text {RLS}$, which are all recommended.

## A numerical illustration using the R-package semTests

We demonstrate the use of the package semTests (Moss, 2024) on a non-normal dataset used in the textbook by Brown (2015, p. 347). This illustration is not intended as a serious data application, but as a simple illustration of the package’s syntax. Also, it shows that using different tests may result in different statistical conclusions. The data have five ordinal variables, each with nine levels, with a sample size of $n=870$. The univariate skewnesses of the variables are 1.5, 2.4, 1.8, 2.2, 3.1, and the univariate excess kurtoses are 1.3, 5.7, 2.3, 4.0, 9.4, indicating moderate non-normality. The model tested is a one-factor model with a residual covariance between two of the variables. We follow Brown (2015) and treat these variables as continuous.

Brown (2015) tests the goodness-of-fit of the model using the SB test. The code in Listing 1 uses the semTests package to perform the goodness-of-fit test. We specify that the package provide *p* values associated with $T_\text {ML}$, SB based on $T_\text {ML}$, and the recommended test for non-nested confirmatory factor models, $\text {pEBA4}_\text {RLS}$.
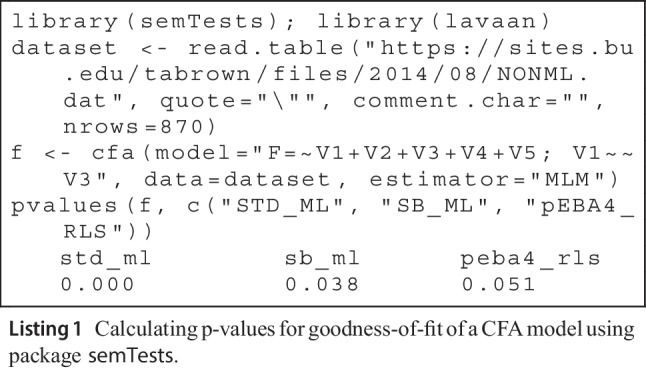


The commonly used SB test indicates that the model is misspecified at the 5% level of significance, while $\text {pEBA4}_\text {RLS}$ *p* value is marginally above this level of significance. The normal-theory ML test has a close to zero *p* value, which is a common finding in empirical work. For non-normal data, this need not indicate a poor fit, as shown in our simulations, where ML rejection rates were highly inflated under correct model specification.

For nested model comparison of two lavaan objects fitted using “MLM” estimation, the syntax of semTests is given in listing 2.
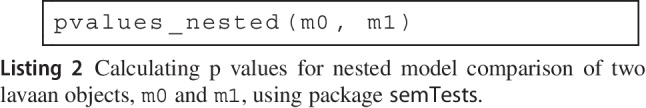


## Discussion and conclusion

Testing model fit in single- and nested-latent-variable models is a common task in social science research. Given that many scientific conclusions in empirical research are partly drawn from such testing, it is of utmost importance to develop and evaluate test procedures that control type I error rates while maintaining adequate power in a variety of settings often encountered in practice, with moderate sample sizes and non-normal data.

Established methods, such as the Satorra–Bentler test and the scaled-and-shifted test, do not always succeed in maintaining type I error control. A class of test procedures to remedy this situation was recently proposed by Foldnes et al. (2025), which outperformed the established tests in a preliminary Monte Carlo study.

### Goodness-of-fit testing

For testing the goodness-of-fit in a confirmatory factor model, our Monte Carlo design extends the model size from 40 observed variables in Foldnes et al. (2025) up to 100 observed variables. To the best of our knowledge, goodness-of-fit testing in such high dimensions has not been evaluated previously, even though these dimensions are practically relevant, e.g., in personality research. We also present the first power analysis of the pEBA procedures.

Our results indicate, echoing the findings of Hayakawa (2019) and Foldnes et al. (2025), that under normality, the RLS chi-square test outperforms the traditional ML chi-square test, especially in high dimensions.

For non-normal data, the robustified tests generally performed better when based on RLS compared to when based on ML. A notable exception is that $\text {pEBA6}^\text {UG}$ narrowly outperforms $\text {pEBA4}_\text {RLS}$ under non-normal data with adequate sample size, but it loses that edge once the sample-size-to-parameters ratio drops. That RLS is preferable as a base statistic also under non-normality might be due to its faster convergence to the limiting distribution under normality also carrying over to non-normal distributions.

As for using the bias-corrected estimator of $\Gamma$, we did not find a general pattern, i.e., it is not generally superior to the non-bias-corrected $\Gamma$ estimator. Surprisingly, for sub-optimal sample-size-to-parameters ratios, the bias-corrected $\Gamma$ estimator can be harmful. A notable exception is the Satorra–Bentler test, which performs best with RLS and unbiased $\Gamma$ estimator, but even then it is overall outperformed by several pEBA procedures. In sum, the bias-corrected $\Gamma$ estimator offered no consistent advantage, and even degraded performance in some conditions.

The most reliable test procedure was $\text {pEBA4}_\text {RLS}$, which consistently ranked at or near the top by RMSE/MAD/ARR across distributions and sizes. Its good performance may be explained by the fact that penalization and block-averaging stabilize the eigenvalue estimates, while RLS stabilizes the base distribution.

As for power, across all families of tests, it increases with sample size and dimension, and declines as distributions deviate from normality. Among the best-performing test procedures in terms of type I error control, the pEBA test outperformed the scaled-shifted test, achieving higher power. At an adequate sample size, all the included pEBA procedures performed similarly in terms of power.

### Nested model testing

A novel contribution is the theoretical extension of pEBA to nested model comparison. We present the first Monte Carlo evaluation of penalized test procedures in this setting, both in terms of type I error and power. We also include modified versions of established tests such as SB and SS based on the unbiased $\Gamma$ estimator and/or $T_\text {RLS}$. In addition, results are provided for two ways of computing $U_D$, either following Satorra (2000) or Satorra and Bentler (2001).

Unexpectedly, nested model testing showed a different pattern of test performance from that observed for goodness-of-fit testing: the ranking of test procedures and their performance differed significantly, and the results were more uniform in the nested model comparison case. The overall winner was the penalized procedure $\text {pEBAdf}^\text {UG}$, which performed uniformly well. Also, one of the established tests, namely SS, performed well. However, SS was found to achieve even better performance when combined with RLS rather than ML.

An important difference between nested model comparison and goodness-of-fit testing in our study pertains to the degrees of freedom of the tests. In the former, the degrees of freedom range from 4 to 133, while in the latter the range is from 80 to 4840.

The sample size conditions from Study 1 were also used in Study 2, though per group, meaning that the total sample size in Study 2 was significantly higher than in Study 1. For these data-generating processes, nested model comparison testing should be less challenging than goodness-of-fit testing, leading to better test performance in the former case, as was observed.

Aggregating across all 189 weak-invariance conditions, the best overall procedure in terms of type I error control is $\text {pEBAdf}^\text {UG}$, which uses ML as base statistic. In general, ML was preferable to RLS as the base statistic when used within pEBA, exhibiting generally higher power and adequate type I error control. However, for the scaled-and-shifted test, it helps to switch from ML to RLS. One conjecture as to why ML is preferred over RLS in conjunction with pEBA methods, is that with lower degrees of freedom, and higher total sample size, ML outperforms RLS under normality.

In contrast to goodness-of-fit testing, for weak invariance testing, we found that the unbiased $\Gamma$ estimator generally improves performance. For instance, $\text {pEBAdf}^\text {UG}$ beats $\text {pEBAdf}$ on RMSE/MAD/ARR; within Satorra–Bentler, the $\text {SB}^\text {UG}$ variant is the best SB option. As for power, $\text {pEBAdf}^\text {UG}$ is within approximately 1 percentage point of $\text {pEBAdf}$ across most conditions, while still being the better test in terms of type I error control. The same pattern appears for other pEBA variants: small power penalties for unbiased $\Gamma$, outweighed by better type I control.

A clear finding is that tests, with the exception of SB, perform better when based on estimating $U_D$ following Satorra (2000), than when following Satorra and Bentler (2001). Our study is the first to extensively evaluate the Satorra (2000) procedure, while previous Monte Carlo studies, to the best of our knowledge, have focused solely on SB, using the approach of Satorra and Bentler (2001). A reason for the lack of studies evaluating the Satorra (2000) procedure is that it, until recently, has not been readily available in software packages (e.g., Brace & Savalei, 2017, p. 470).

The Satorra and Bentler (2001) testing procedure is currently the default method in lavaan for nested model comparison under non-normality. In the simulation setup in the present article, this test performs poorly. The default test in Mplus is SS, which performs relatively better, although not as well as $\text {pEBAdf}^\text {UG}$.

### Practical recommendations

Our practical recommendation for goodness-of-fit testing in confirmatory factor modeling is to use $\text {pEBA4}_\text {RLS}$, which was also recommended by Foldnes et al. (2025).

For nested model comparisons, our recommended test is $\text {pEBAdf}^\text {UG}$.

### Limitations and further research

As in any simulation study, our results are contingent upon the conditions included. Although we span a range of six non-normal distributional classes, our results need not replicate over other multivariate distributions than those included. Additionally, we employed distributions with identical skewness and kurtosis across all variables, which is unlikely to hold exactly in empirical situations.

The same argument pertains to the model types included. For instance, we included a specific five-factor model for goodness-of-fit test evaluations and a one-factor model for nested model comparisons. Future research should extend these simulations to model additional configurations. Also, we included only one type of nested model comparison, namely weak measurement invariance.

The test procedures investigated are applicable to a much larger set of models, e.g., structural equation models. We consider evaluating the potential of the penalized procedures in a larger model class a worthy topic for future research. Another topic for future research is to investigate how small the sample size can be for RLS to still perform well under normality. In our study, we did not find any normal conditions where RLS performed poorly. Similarly, nested model comparison performance was overall satisfactory in our study, even in our most difficult simulation setup with a sample size of 400, 20 observed variables, and eight groups. It is therefore of interest to perform future investigations into how small a sample size is admissible for $\text {pEBAdf}^\text {UG}$ to perform well.

Two further limitations are that we did not include either missing data or ordinal-categorical data. Since missing data inference is similarly structured to the complete data case (Yuan & Bentler, 2000), the penalized test procedures may be extended to handle missingness. Similarly, the pEBA procedures may also handle ordinal-categorical data as also here, the test statistics become mixtures of chi-square variates. We consider these topics worthy of future research.

A final remark is that there is a much larger class of models where test statistics converge in distribution to a mixture of chi-square variates (Hansen, 2021). The penalization framework may also improve inference quality in such settings, especially in high dimensions.

### Conclusion

We have conducted an extensive Monte Carlo study with 525 conditions to evaluate 38 test procedures for goodness-of-fit and nested model comparisons in confirmatory factor models. The overall best-performing tests (pEBA with four blocks based on RLS for goodness-of-fit and pEBA with singleton blocks and unbiased $\Gamma$ estimator for nested model comparisons) belonged to the penalized eigenvalue block averaging class of tests recently proposed by Foldnes et al. (2025), outperforming established tests such as the Satorra–Bentler and the scaled-and-shifted tests, both in terms of type I error control and power.

## Data Availability

The data and materials for all simulations are available at https://osf.io/h2y3n/.

## Code availability

Source code is openly available at https://CRAN.R-project.org/package=semTests.

## Declarations

### Open Practices Statement

The data and materials for all simulations are available at https://osf.io/h2y3n/.
